# Novel parameters for predicting fluid responsiveness during the mini fluid challenge and ability of the cardiac power index: an observational cohort study

**DOI:** 10.55730/1300-0144.5688

**Published:** 2023-08-26

**Authors:** Taner ABDULLAH, Hürü Ceren GÖKDUMAN, İşbara Alp ENİŞTE, Ayşe Gülşah ATASEVER, Achmet ALİ, Funda GÜMÜŞ ÖZCAN

**Affiliations:** 1 Department of Anesthesiology, İstanbul Başakşehir Çam and Sakura City Hospital, İstanbul, Turkiye; 2 Department of Anesthesiology, University Hospitals of the KU Leuven, Leuven, Belgium; 3 Department of Anesthesiology and Reanimation, İstanbul Medical Faculty, İstanbul University, İstanbul, Turkiye

**Keywords:** Intraoperative monitoring, fluid therapy, positive-pressure respiration, stroke volume

## Abstract

**Background/aim:**

The percentage change in the stroke volume index (SVI) due to the mini fluid challenge (MFC) (MFC-ΔSVI%) is used commonly in daily practice. However, up to 20% of patients remain in the gray zone of this variable. Thus, it was aimed to compare the MFC-ΔSVI% and the percentage change in the cardiac power index (CPI) due to the MFC (MFC-ΔCPI%) with the baseline values of the pulse pressure variation (PPV) and stroke volume variation (SVV) in terms of their abilities to predict fluid responsiveness.

**Materials and methods:**

The SVI, CPI, SVV, and PPV were recorded before 100 mL of isotonic saline was infused (MFC), after MFC was completed, and after an additional 400 mL of isotonic saline was infused to complete 500 mL of fluid loading (FL). Patients whose SVI increased more than 15% after the FL were defined as fluid responders.

**Results:**

Sixty-seven patients completed the study and 35 (52%) of them were responders.The areas under the receiver operating characteristics curves for the MFC-ΔSVI% and MFC-ΔCPI% (0.94; 95% CI: 0.86–0.99 and 0.89; 95% CI: 0.79–0.95, respectively) were significantly higher than those for the SVV and PPV (0.63; 95% CI: 0.50–0.75 and 0.55; 95% CI: 0.42–0.67, respectively) (p < 0.001 for all of the comparisons). The gray zone analysis revealed that the MFC-ΔSVI% values of 12 patients were in the gray zone. Of the 12, the MFC-ΔCPI% values of 7 patients were outside of the gray zone.

**Conclusion:**

Fluid responsiveness can be predicted more accurately using the MFC-ΔSVI% and MFC-ΔCPI% than using the SVV and PPV. Additionally, concomitant use of the MFC-ΔSVI% and MFC-ΔCPI% is recommended, as this approach diminishes the number of patients in the gray zone.

## 1. Introduction

The titration of fluid therapy is a fundamental part of the intraoperative period [1]. Both hypovolemic and hypervolemic states increase the risk of postoperative complications [2]. Therefore, while optimizing the stroke volume (SV), it is crucial to avoid fluid challenges that will not result in an SV increment [3]. In this context, evaluating fluid responsiveness is highly recommended by the perioperative guidelines [4].

The dynamic indices derived from cardiopulmonary interactions, such as pulse pressure variation (PPV) and SV variation (SVV), have been shown to be superior to the static preload parameters in terms of predicting fluid responsiveness [5, 6]. However, several conditions limit their ability, including the use of a tidal volume below 8 mL/kg of ideal body weight (IBW) [7]. The mini fluid challenge (MFC) is one of the functional hemodynamic tests (FHTs) that was developed for such conditions where the PPV and SVV are not applicable [4, 8]. This test depends on evaluating the hemodynamic changes resulting from a rapid infusion of 100 mL of crystalloids [8]. The absolute or percentage change in the stroke volume index (SVI) due to the MFC (MFC-ΔSVI and MFC-ΔSVI%, respectively) are the 2 most frequently analyzed variables when an arterial waveform analysis device is used [8–12]. Although the sensitivity and specificity of these variables are satisfying, up to 20% of patients remain in the gray zone, where the diagnosis is uncertain [11, 12]. Therefore, there is a need for alternative parameters while using devices analyzing the arterial waveform in order to improve the predictive ability of the MFC.

From this point of view, the cardiac power index (CPI) is a promising parameter, as it is correlated with stroke work and the right ventricle end-diastolic volume [13, 14]. To the best of our knowledge, there are no studies that have evaluated the ability of the percentage change in the CPI due to the MFC (MFC-ΔCPI%) to predict fluid responsiveness.

The primary aim of this study was to compare the abilities of the MFC-ΔSVI% and MFC-ΔCPI% with the baseline PPV and SVV to predict fluid responsiveness in patients who are undergoing major abdominal surgery with laparotomy and are ventilated with 6–8 mL/kg IBW tidal volumes (TVs). The secondary aims were to evaluate other variables derived from arterial waveform analysis for the same outcome and determine the prognostic indices (i.e., the best cut-off values, sensitivity, and specificity percentages) for all of the variables.

## 2. Materials and methods

### 2.1. Study design and patient selection

This study was designed as a single-center prospective observational study and performed in line with the principles of the Declaration of Helsinki. Ethical approval was obtained from the Clinical Research Ethics Committee of İstanbul Başakşehir Çam and Sakura City Hospital (no.: 2022.02.51, dated: February 2022). The inclusion criteria were as follows: age at least 18 years, elective abdominal surgery with laparotomy under general anesthesia, and the use of invasive blood pressure monitoring. Patients were enrolled in the study between March and June 2022, and written informed consent was obtained from all of the patients in the preoperative period. Patients with any of the following conditions were excluded: body mass index (BMI) >35 kg/m^2^, preoperative arrhythmia, left ventricle ejection fraction <50%, systolic peak velocity of tricuspid annular motion <0.17 m/s, static respiratory system compliance (Crs) <35 mL/cmH_2_O, moderate or severe valvular heart disease, chronic pulmonary disease, chronic medication with beta-blockers, and ASA score >3. While applying the protocol, patients with the following conditions were also excluded: new onset bleeding or arrhythmia, loss of the quality of arterial signal, and need for hemodynamic intervention.

### 2.2. Anesthesia management

The peripheral oxygen saturation, heart rate (HR), noninvasive blood pressure, and bispectral index (BIS monitor; Medtronic, Brooklyn Park, MN, USA) values of the patients were monitored following their arrival at the operating room. Anesthesia was induced with 1% propofol along with 1 mcg/kg fentanyl and 0.6 mg/kg rocuronium bromide and maintained with sevoflurane (1%–2%) and remifentanil (0.05–0.3 mcg/kg/min), aiming BIS values between 40 and 60. Mechanical ventilation included volume-controlled ventilation (Perseus A500, Drager, Lübeck, Germany) with a TV of 6–8 mL/kg IBW at a rate of 12–15/min, and an inspiratory-to-expiratory ratio of 1/2 in 40% oxygen and air with a positive end-expiratory pressure (PEEP) of 5–7 cmH_2_O. The IBW was calculated using Robinson’s formula [15]. Fluid administration during the surgery was set between 3–5 mL/kg/h by the attending anesthesiologist, it was stopped during the application of the study protocol and then resumed upon completion of the protocol. The decision to administer 500 mL of fluid loading (FL) was at the discretion of the attending anesthesiologist and was not standardized by a protocol.

### 2.3. Respiratory and hemodynamic monitoring

Prior to the start of ventilation with the anesthesia machine, the Crs values of the patients were automatically calculated by a ventilator (Hamilton-C1 ventilator; Hamilton Medical, Bonaduz, Switzerland) capable of applying expiratory and inspiratory hold maneuvers.

The left radial artery was catheterized after the induction of anesthesia. An arterial catheter (Vygon, Padova, Italy) dedicated to radial artery catheterization and the analysis of arterial waveform via a MostCare monitor (Vygon) was used, and it was attached to the pressure transducer of the aforementioned device. The square-wave test was used to ensure the absence of overdamping and underdamping of the arterial pressure wave.

### 2.4. Parameters of the arterial waveform analysis

The MostCare monitor analyzes the arterial waveform with a sampling rate of 1000 points/s [16]. This feature allows it to determine the points of instability profile of the arterial waveform and calculate the beat-to-beat SVI [17]. Systolic, diastolic, and dicrotic pressure (DicP) points, mean arterial pressure (MAP), and pulse pressure are also identified directly from the arterial wave analysis. Subsequently, several hemodynamic parameters are calculated automatically, as follows:


$$\begin{array}{l} \text{CPI }(\text{W}/{\text{m}}^{2}):\text{CI}\times \text{MAP}/451\\ \text{SVV}(\%):\;(\text{SVmax}-\text{SVmin})/\text{SVmean }(\text{calculated every }30\;\text{s})\\ \text{PPV}(\%):(\text{PPmax}-\text{PPmin})/\text{PPmean }(\text{calculated every }30\;\text{s})\\ \text{Arterial elastance},\;\text{Ea }(\text{mmHg}/\text{mL}):\text{DicP}/\text{SV} \end{array}$$

The maximal slope of the systolic portion of the arterial pressure waveform (maximal pressure / time ratio, dP/dt_max_): Obtained directly from the sampling of the arterial wave.

Cardiac cycle efficiency (CCE): Calculated using the relevant equation [17].

### 2.5. Protocol

The study protocol was applied following the confirmation of the hemodynamic stability during the surgery (defined as the mean arterial pressure (MAP) change <10% for 3 min). BIS values were between 40 and 60 and within ±10% of the baseline value in all of the patients during the protocol. The surgical team was also warned not to apply a new onset surgical stimulus. Only the first sets of measurements were recorded for each patient.

The hemodynamic parameters were recorded at 3 time points (T1–T3). The T1 measurement was performed before 100 mL of isotonic saline was infused over 1 min (MFC). A minute after the MFC was completed, the T2 measurement was performed. Finally, the T3 measurement was performed 3 min after an additional 400 mL of isotonic saline was infused within 10 min to complete 500 mL of FL. Patients whose SVI showed an increase of more than 15% after the FL (FL-ΔSVI% > 15) were classified as responders. Absolute changes of the parameters due to MFC were calculated as follows and defined with the MFC-ΔXXX sign (i.e., MFC-ΔSVI, MFC-ΔCPI): SVI(T2) – SVI(T1), CPI(T2) – CPI(T1), PPV(T1) – PPV(T2), SVV(T1) – SVV(T2), Ea(T1) – Ea(T2), dP/dTmax(T2) – dP/dTmax(T1), CCE(T2) – CCE(T1). The percentage changes of the parameters due to the MFC were calculated as the ratio between the absolute change and baseline (T1) values for each parameter and indicated as MFC-ΔXXX% (i.e., MFC-ΔSVI%, MFC-ΔCPI%).

### 2.6. Statistical analysis

In view of previous results, the area under the receiver operating characteristics curve (ROCAUC) of the baseline PPV and SVV were expected to be <0.65 [11, 18]. Although the ROCAUC of the MFC-ΔSVI% is usually >0.90 in the literature [8], considering the fact that the MFC-ΔCPI% has not been studied previously, a ROCAUC value of >0.85 was assumed for the MFC-ΔSVI% and MFC-ΔCPI%. Assuming that at least 40% of the patients would be fluid responsive, it was calculated that at least 65 patients were needed to reveal such a difference (type I error of 5% and type II error of 20%).

The distribution of the interval data was evaluated using the Shapiro-Wilk test. Normally distributed data were presented as the mean ± standard deviation, and nonnormally distributed data were presented as the median (25th–75th percentile). Categorical data were presented as the number and frequency. The hemodynamic parameters of the responders and nonresponders were compared with the student t test or Mann-Whitney U test, whereas the hemodynamic changes within the groups during the MFC were analyzed using the repeated measurements 1-way analysis of variance (ANOVA) or Friedmann test, as appropriate. The Bonferroni adjustment was applied for pairwise comparisons. The relationships between the MFC-ΔSVI% and FL-ΔSVI%, and between the MFC-ΔCPI% and FL-ΔSVI% were evaluated with the Spearman correlation analysis. Receiver operating characteristics (ROC) curves were created in order to evaluate the ability of the baseline values and absolute and percentage changes due to the MFC of the following parameters to predict fluid responsiveness: the SVI, CPI, SVV, PPV, Ea, dP/dt_max_, and CCE. The ROCAUCs of the MFC-ΔSVI% and MFC-ΔCPI% were compared with those of the baseline PPV and SVV with the approach defined by DeLong et al. [19]. Cut-off values for the variables and their sensitivity and specificity values were calculated using the Youden index (sensitivity + specificity – 1). Statistical significance was accepted as p < 0.05.

The gray zone analysis was performed for the MFC-ΔSVI% and MFC-ΔCPI% as described by Coste et al. [20]. The upper and lower cut-off points determining the gray zone were defined with the values associated with a positive likelihood ratio =0.1, ensuring a posttest probability <0.05 and a negative likelihood ratio =10, ensuring a posttest probability >0.90, respectively.

Statistical analyses were performed using IBM SPSS Statistics for Windows 21.0 (IBM Corp., Armonk, NY, USA) or MedCalc 16.1 (MedCalc Software Ltd, Ostend, Belgium), as appropriate.

## 3. Results

### 3.1. Patient characteristics and hemodynamic data

**Figure 1:**
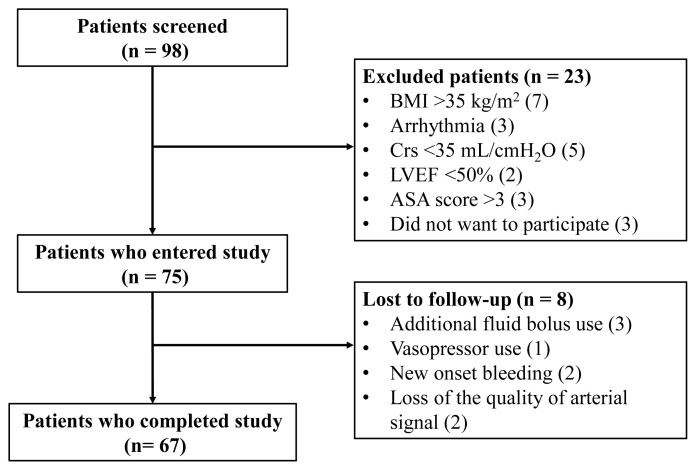
Study flow chart. BMI: body mass index, Crs: static respiratory system compliance, LVEF: left ventricle ejection fraction, ASA score: American Society of Anesthesiologists score.

Of the 75 patients who participated in this study, 67 completed the protocol (Figure 1). Patient characteristics are shown in Table 1. Of these patients, 35 (52%) were responders, and 32 (48%) were nonresponders to FL. The HR, MAP, SVI, CPI, Ea, dP/dt_max_, CCE, PPV, and SVV values of the patients during the T1, T2, and T3 measurement times are shown in Table 2.

### 3.2. Change in the SVI and CPI after MFC in the responders and nonresponders

A higher percentage change in the SVI and CPI was observed among the responders after the MFC (p < 0.001 for both). The MFC-ΔSVI% values were 11.5 (7.7–18.2) and 3.0 (0–5.6), while the MFC-ΔCPI% values were 17 (7.5–26.5) and 0 (−4.2–4.2) in the responders and nonresponders, respectively.

### 3.3. Change in the SVI after FL in the responders and nonresponders and correlation with the MFC-ΔSVI% and MFC-ΔCPI%

A higher percentage change was observed in the SVI among the responders after FL (p < 0.001). The FL-ΔSVI% values were 27.3 (20–44.8) and 6.7 (3.1–11.3) in the responders and nonresponders, respectively.

The MFC-ΔCPI% was moderately correlated with the FL-ΔSVI% (r = 0.66, p < 0.001), while there was a strong correlation between the MFC-ΔSVI% and FL-ΔSVI% (r = 0.83, p < 0.001).

### 3.4. Predicting fluid responsiveness

**Figure 2:**
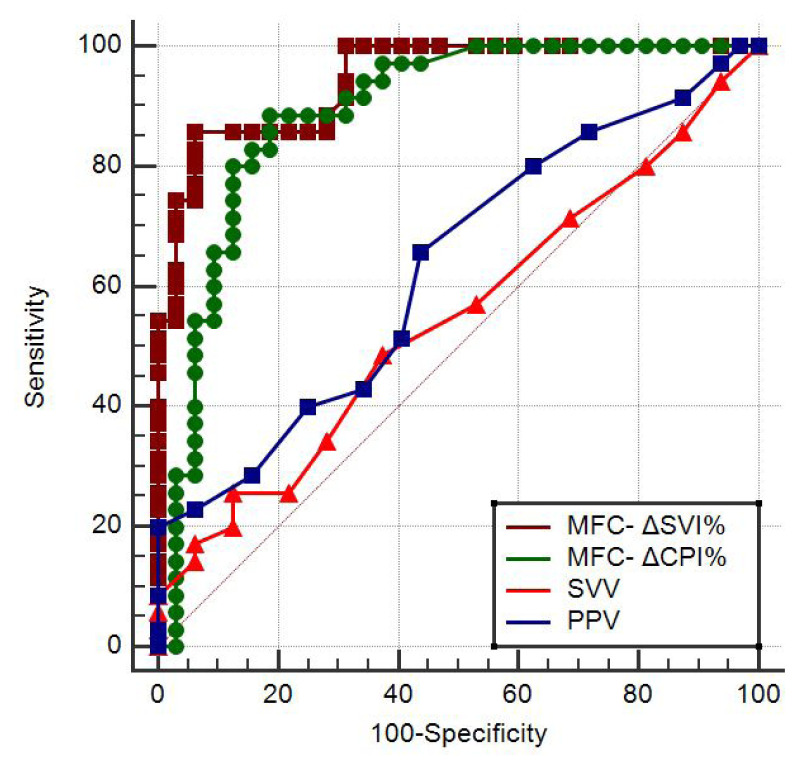
ROC curves generated for the MFC-ΔSVI%, MFC-ΔCPI%, SVV, and PPV showing the ability to predict fluid responsiveness.

ROC curves were created to determine the ability of the baseline values and the absolute and percentage changes due to MFC of the following parameters to predict fluid responsiveness: the SVI, CPI, SVV, PPV, Ea, dP/dt_max_, and CCE (Table 3). As the primary outcome, the ROCAUCs of the MFC-ΔSVI% and MFC-ΔCPI% (0.94; 95% CI: 0.86–0.99 and 0.89; 95% CI: 0.79–0.95, respectively) were statistically significantly higher than those of the PPV and SVV (0.63; 95% CI: 0.50–0.75 and 0.55; 95% CI: 0.42–0.67, respectively) (p < 0.001 for all of the comparisons) (Figure 2). There was no statistically significant difference between the ROCAUCs of the MFC-ΔSVI% and MFC-ΔCPI% (p = 0.24). The best cut-off values and diagnostic performances of the variables are shown in Table 3.

**Figure 3:**
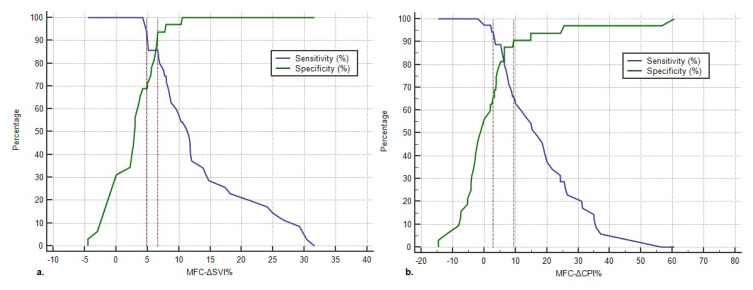
Gray zones of the MFC-ΔSVI% and MFC-ΔCPI%. **a.** Gray zone of the MFC-ΔSVI%. The lower cut-off point is 4.8% (with 94.3% sensitivity). The upper cut-off point is −6.67% (with 93.8% specificity). The gray zone includes 12 (17.9%) patients. **b.** Gray zone of the MFC-ΔCPI%. The upper cut-off point is 9.4% (with 90.6% specificity). The lower cut-off point is 2.9% (with 94.3% sensitivity). The gray zone includes 18 (26.9%) patients.

A gray zone analysis was conducted for the MFC-ΔSVI% and MFC-ΔCPI%. The gray zone thresholds for the MFC-ΔSVI% were 4.8% and 6.67%, and 12 patients (17.9%) were inside of the gray zone (Figure 3a). The gray zone thresholds for the MFC-ΔCPI% were 2.9% and 9.4%, and 18 patients (26.9%) were inside of the gray zone (Figure 3b).

The MFC-ΔCPI% values of the 12 patients who were inside of the gray zone of the MFC-ΔSVI% were further evaluated. Of these 12 patients, 7 were outside of the gray zone of the MFC-ΔCPI% and were accurately diagnosed with this variable, While 3 patients were inside of the gray zone of the MFC-ΔCPI%, even though the predictions of the MFC-ΔSVI% and MFC-ΔCPI% for these patients were in concordance and accurate. The last 2 patients were also inside of the gray zone of both variables. However, there was a discordance between the predictions of these variables that favored the MFC-ΔSVI% (Table 4).

## 4. Discussion

The main findings of this study showed that the MFC-ΔSVI% and MFC-ΔCPI% predict fluid responsiveness with high sensitivity and specificity and better than the SVV and PPV in patients who are undergoing laparotomy and are ventilated with TVs of 6–8 mL/kg IBW. Although the gray zone analysis revealed that 17.9% of the patients were diagnosed inconclusively with the MFC-ΔSVI% when the MFC-ΔCPI% was used as the additional parameter, 58% of these patients were saved from the gray zone and diagnosed accurately.

The OPTIMIZE trial revealed that the abilities of these indices to predict fluid responsiveness are also impaired in patients undergoing laparotomy, even if the patients are ventilated with 8 mL/kg IBW of TVs [21]. Based on a study conducted on critically ill patients [18], one can argue that the absolute or percentage changes of the PPV and SVV due to the MFC might have a reasonable diagnostic performance (ROCAUCs for the MFC-ΔPPV and MFC-ΔSVV: 0.92 and 0.91, respectively) [18]. However, the results of the current study are inconsistent with this finding, as it was found that the ROCAUC values of absolute and percentage changes of the PPV and SVV due to the MFC were all ≤0.70. This inconsistency can be explained by the impaired effect of cardiopulmonary interactions on the right ventricle preload due to the loss of the transdiaphragmatic pressure after laparotomy since the transdiaphragmatic pressure works as a driving force for the blood through inferior vena cava towards the right ventricle [22]. Another study also supports the attenuating effect of the reduced abdominal pressure on the right ventricle preload changes caused by cardiopulmonary interactions, as they found a reduction in the SVV and PPV (50% and 40%, respectively) following laparotomy [23]. Consequently, neither the baseline values nor the MFC-induced changes of these parameters are suitable to use for predicting fluid responsiveness in patients undergoing open abdominal surgery.

The MFC has a unique place among FHTs because it is independent of cardiopulmonary interactions [24]. Therefore, this method has the ability to predict fluid responsiveness in patients with spontaneous breathing [25], low Crs, and a high BMI value [10], and in patients ventilated with TVs <8 mL/kg IBW [9, 12]. Messina et al. evaluated 7 MFC studies with 368 fluid challenges and found a pooled ROCAUC of 0.91 with a cut-off of 5% for the MFC-ΔSVI% [8]. The results of this present study are in agreement with their findings.

Using the MFC-ΔSVI% during the MFC is widely recognized in the literature, as mentioned above. However, in line with the results herein, previous studies revealed that up to 20% of patients cannot be diagnosed accurately as they are placed in the gray zone of the MFC-ΔSVI%, a range where it is likely to end up with a false positive/negative diagnosis [11, 12]. To diagnose these patients accurately, other parameters derived from the arterial waveform analysis were evaluated, including the CPI, dP/dt_max_, Ea, and CCE during the MFC. The MFC-ΔCPI% was the variable that showed a remarkable performance with a ROCAUC value of 0.89. Of the 12 (17.9%) patients who were in the gray zone of the MFC-ΔSVI%, 7 were diagnosed accurately with the MFC-ΔCPI% values out of the gray zone. With the support of the CPI, the population in the gray zone of the MFC-ΔSVI% was reduced by 58.3%. To our knowledge, this is the first study to have shown the ability of the MFC-ΔCPI% to predict fluid responsiveness in any patient group. In addition, there are no studies demonstrating that the gray zone area can be minimized using a second arterial waveform analysis parameter. The CPI is defined as the product of the cardiac index, MAP, and a conversion factor to convert the units to watts (W) [25]. While the strong correlation between the CPI and stroke work (area under the volume-pressure loop of the cardiac cycle) is already well-known [13], it has been shown that there is also a correlation between the CPI and the end-diastolic volume of the right ventricle [14]. From this standpoint, the diagnostic accuracy of the MFC-ΔCPI% is not surprising, since both of the aforementioned parameters are expected to increase concomitantly in responders.

The other parameters evaluated in this study were the CCE, Ea, and dP/dTmax. The CCE describes the relation between the stroke work generated and the total energy consumed for providing that stroke work during a cardiac cycle [17]. Stroke work is formed as a result of the interactions between cardiovascular elements such as the contractility, preload, and arterial load [26]. Therefore, a low/negative CCE value reflects the presence of a derangement in at least one of these elements [17]. If a low/negative CCE is due to the decreased preload, an increase in the CCE is expected after the FL in responders [27]. Regarding the Ea, this parameter is an integrative index of arterial load that consists of systemic vascular resistance, total arteria elastance, and characteristic impedance [28]. Because an increase in the SVI is usually followed by a decrease in the systemic vascular resistance, the Ea is also expected to decrease following a FL in responders [29]. Finally, the dP/dTmax is a parameter mainly determined by contractility and arterial load [30]. However, this parameter might also be affected from the preload status, as Garcia et al. reported a decrement in the dP/dTmax following acute preload reduction [31]. Therefore, an increase in the dP/dTmax might be expected in responders after FL.

Even though all of these parameters revealed the expected changes in the responders in the current study, the explained contribution of the contractility and arterial load to these parameters probably prevented them from demonstrating satisfying diagnostic accuracy. It has already been revealed that baseline CCE and Ea values demonstrate insufficient accuracy in terms of predicting fluid responsiveness (ROCAUC values: 0.77 and 0.59, respectively) [27]. However, to our knowledge, this is the first study to report the ability of the baseline dP/dTmax and the abilities of the MFCΔ and MFCΔ% values of the dP/dTmax, CCE, and Ea to predict fluid responsiveness.

This study had several limitations. First, the infusion rate of the MFC and total FL were 100 mL in 1 min and 500 mL in 10 min, respectively. Different infusion rates and times may result in different outcomes. Second, the Mostcare monitor was used, which is an uncalibrated device for evaluating arterial waveform. The use of externally and internally calibrated alternative devices may result in different cut-off and diagnostic performance values. Third, this study was conducted on patients undergoing major abdominal surgery with laparotomy and in the supine position. The results should be extrapolated carefully for different clinical scenarios.

To conclude, in patients undergoing open abdominal surgery, fluid responsiveness could be predicted more accurately using the MFC-ΔSVI% and MFC-ΔCPI% than using the SVV and PPV. Additionally, concomitant use of the MFC-ΔSVI% and MFC-ΔCPI% is recommended, as this approach diminishes the number of patients in the gray zone.

## Figures and Tables

**Table 1:** Characteristics of the patients.

Variables (n = 67)	

Gender (male/female)	37/30

Age (years)	54.3 ± 11.6

BMI (kg/m^2^)	25.3 (23.0–28.1)

IBW (kg)	59.2 (55.4–68.9)
PEEP (cm H_2_O)	5 (5–5)

TV (mL)	450 (400–500)

Driving Pressure (cm of H_2_O)	9 (8–11)

TV (mL/kg of IBW)	7.5 (7.1–7.9)

Static compliance (mL/cm of H_2_O)	50 (39.1–60)

Values are expressed as the number, mean ± SD, median (25th to 75th percentile). BMI: body mass index, IBW: ideal body weight, PEEP: positive end-expiratory pressure, TV: tidal volume.

**Table 2:** Hemodynamic variables at baseline, and after 100 and 500 mL of FL.

	Baseline (T1)	After 100 mL of fluid (T2)	After 500 mL of fluid (T3)	p-value
HR (beats/min)				
Responders	78 ± 12.5	76.3 ± 12.6*	74.9 ± 13*†	<0.001
Nonresponders	73.3 ± 12.8	72.3 ± 12.5	72.4 ± 11.1	0.19
P intergroup	0.13			
MAP (mmHg)				
Responders	74.6 ± 12.2	78.2 ± 13.3*	85.5 ± 15.3*†	<0.001
Nonresponders	77 ± 13	77.5 ± 12.8	80.3 ± 11.7*	0.002
P intergroup	0.43			
SVI (mL/m^2^)				
Responders	28.5 ± 7.7	32.3 ± 8.3*	37.5 ± 8.8*†	<0.001
Nonresponders	33.7 ± 6.3	34.8 ± 7.0*	36.2 ± 7.2*†	<0.001
P intergroup	0.004			
CPI (W/m^2^)				
Responders	0.35 ± 0.09	0.42 ± 0.11*	0.51 ± 0.13*†	<0.001
Nonresponders	0.42 ± 0.09	0.42 ± 0.09	0.48 ± 0.12*†	<0.001
P intergroup	0.006			
CCE				
Responders	−0.06 (−0.38–0.07)	0.01 (−0.32–0.16)*	0.04 (−0.15–0.17)*	<0.001
Nonresponders	−0.06 (−0.24–0.22)	0.01 (−0.17–0.21)	0.02 (−0.30–0.19)†	0.03
P intergroup	0.17			
Ea (mmHg/mL)				
Responders	1.46 (1.26–2.00)	1.44 (1.09–1.79)*	1.27 (1.04–1.72)*	<0.001
Nonresponders	1.26 (1.05–1.59)	1.27 (1.01–1.54)	1.30 (1.11–1.58)	0.15
P intergroup	0.04			
dP/dt_max_ (mmHg/ms)				
Responders	0.85 (0.69–0.97)	0.92 (0.72–1.03)*	0.98 (0.78–1.11)*	<0.001
Nonresponders	0.88 (0.75–0.99)	0.88 (0.74–1.08)	0.95 (0.80–1.15)*	0.01
P intergroup	0.42			
PPV (%)				
Responders	10 (8–13)	8 (6–10)*	6 (4–7)*†	<0.001
Nonresponders	8 (6–12)	7 (5–10) *	5 (3–7)*†	<0.001
P intergroup	0.07			
SVV (%)				
Responders	7 (5–11)	9 (7–11)*	6 (4–9)* †	<0.001
Nonresponders	7 (5–9)	8 (6–11)	6 (4–10)* †	<0.001
P intergroup	0.47			

Values are expressed as the mean ± SD or median (25th to 75th percentile). P intergroup: comparison between the responders and nonresponders with the student t test or Mann-Whitney U test. P-values = comparison of different time points within the groups with the repeated measurements 1-way ANOVA or Friedman test.

* Significant difference compared with the T1 value following the Bonferroni adjustment (p < 0.016).

† Significant difference compared with the T2 value following the Bonferroni adjustment (p < 0.016).

HR: heart rate, MAP: mean arterial pressure, SVI: stroke volume index, CPI: cardiac power index, CCE: cardiac cycle efficiency, Ea: arterial elastance, dP/dt_max_: maximal pressure/time ratio, PPV: pulse pressure variation, SVV: SV variation.

**Table 3:** Best cut-off values and diagnostic performances of the variables.

Variable	ROCAUC (95% CI)	Best cut-off	Sensitivity (%)	Specificity (%)
MFC-ΔSVI%	0.94 (0.86–0.99)	>6.67	86	94
MFC-ΔCPI%	0.89 (0.79–0.95)	>5.3	89	81
PPV	0.63 (0.50–0.75)	>8	66	56
SVV	0.55 (0.42–0.67)	>10	26	88
MFC-ΔSVI	0.88 (0.78–0.95)	>1	94	66
MFC-ΔCPI	0.87 (0.77–0.94)	>0.02	77	84
CPI	0.72 (0.60–0.82)	≤0.33	57	78
MFC-ΔEA	0.72 (0.60–0.82)	>0.05	69	75
MFC-ΔEA%	0.70 (0.58–0.81)	>4.9	60	81
MFC-ΔPPV%	0.70 (0.57–0.80)	>18.18	63	72
MFC-ΔPPV	0.70 (0.58–0.81)	>1	66	66
SVI	0.69 (0.57–0.80)	≤29	63	84
MFC-ΔDP/DT%	0.68 (0.56–0.79)	>2.33	77	59
EA	0.65 (0.52–0.76)	>1.32	71	63
MFC-ΔCCE	0.64 (0.52–0.76)	>0.05	54	72
CCE	0.60 (0.47–0.72)	≤0.09	86	41
DP/DT	0.56 (0.43–0.68)	≤0.62	20	97
MFC-ΔSVV	0.56 (0.44–0.68)	>2	20	91
MFC-ΔSVV%	0.54 (0.41–0.66)	>10	57	53
MFC-ΔDP/DT	0.69 (0.56–0.79)	>0.01	80	56

The best cut-off values were determined using the Youden index (J = sensitivity + specificity − 1). ROCAUC: are under the receiver operating characteristics curve, MFC-ΔSVI%: percentage change in the SVI due to the mini fluid challenge (MFC), MFC-ΔCPI%: percentage change in the CPI due to the MFC, MFC-ΔXXX: absolute change in the aforementioned parameter due to the MFC, MFC-ΔXXX%: percentage change in the aforementioned parameter due to the MFC.

**Table 4:** MFC-ΔSVI% and MFC-ΔCPI values of the patients inside the gray zone of the MFC-ΔSVI%.

Case no.	Actual fluid status	Responder according to the MFC-ΔSVI%	Responder according to the MFC-ΔCPI%	Inside the gray zone of the MFC-ΔCPI%	MFC-ΔSVI%	MFC-ΔCPI%
49	Responder	No	Yes	No	4.8	11.6
8	Responder	No	Yes	No	4.88	47.37
18	Responder	No	Yes	No	5	10
27	Nonresponder	No	No	No	5	−8.11
34	Responder	No	Yes	No	5.1	31.3
14	Nonresponder	No	Yes	Yes	5.56	6.52
33	Nonresponder	No	No	Yes	5.6	2.9
17	Nonresponder	No	No	No	6.06	0
15	Nonresponder	No	No	No	6.25	−2.78
7	Nonresponder	No	No	Yes	6.45	2.13
4	Nonresponder	No	No	Yes	6.67	3.77
25	Nonresponder	No	Yes	Yes	6.67	6.45

Patients indicated with red are outside the gray zone of the MFC-ΔCPI%. Patients indicated with blue are inside the gray zone of the MFC-ΔCPI%, even though the diagnosis of the MFC-ΔCPI% is in line with the diagnosis of the MFC-ΔSVI%.
